# Human Papillomavirus-Related Anal Precancerous Lesions in Men Who Have Sex With Men: A Comparative Study of Pre-exposure Prophylaxis Users and HIV-Positive Individuals

**DOI:** 10.1093/cid/ciaf724

**Published:** 2026-01-05

**Authors:** Magali Surmont, Michel Verheyden, Shaira Sahebali, Mathijs Goossens, Sabine Allard, Sebastien Kindt

**Affiliations:** Department of Gastroenterology and Hepatology, UZ Brussel, Brussels, Belgium; Vrije Universiteit Brussel (VUB), Brussel, Belgium; Department of Dermatology, UZ Brussel, Brussels, Belgium; Department of Pathology, UZ Brussel, Brussels, Belgium; Centrum Voor Kankeropsporing, Brugge, Belgium; Department of Infectious Diseases, UZ Brussel, Brussels, Belgium; Department of Gastroenterology and Hepatology, UZ Brussel, Brussels, Belgium; Vrije Universiteit Brussel (VUB), Brussel, Belgium

**Keywords:** HPV, anal precancerous lesion, MSM, HIV, PrEP

## Abstract

**Background:**

Although anal cancer is rare, it disproportionately affects high-risk groups such as men who have sex with men (MSM) and people with human immunodeficiency virus (HIV). MSM using Pre-Exposure Prophylaxis (MSM-PrEP) represent an emerging subgroup with behavioral risk factors that may increase susceptibility to High-Risk Human Papillomavirus (HRHPV) infection and anal High-grade Squamous Intraepithelial Lesions (HSIL). However, data on precancerous anal lesions in this group remain unexplored.

This study aims to compare the prevalence and distribution of histological abnormalities detected by High-Resolution Anoscopy (HRA) between MSM-PrEP and MSM with HIV (HIV + MSM) and to explore the association between anal HSIL and anal swab results using cytology and HRHPV testing.

**Methods:**

This secondary exploratory analysis extended a prospective, monocentric cross-sectional study on cytology and HPV infection conducted at the HIV Reference Centre and PrEP Clinic of Universitair Ziekenhuis Brussel by incorporating histological data.

**Results:**

A total of 85 MSM-PrEP and 93 HIV + MSM underwent HRA. Histologically confirmed anal HSIL did not differ significantly between MSM-PrEP and HIV + MSM (OR 0.6, 95% CI .32–1.25). No significant variation was observed in the overall histological outcome distribution (*P* = .401). A history of gonorrhea was significantly associated with HSIL (OR 2.39, 95% CI 1.03–5.55). Cytology showed limited discriminatory value, while HRHPV infection was present in all HSIL cases. Many lesions were linked to HRHPV types other than HPV16.

**Conclusions:**

MSM-PrEP may be at similarly high risk for anal HSIL as HIV + MSM, underscoring the need for tailored screening strategies.

## Teaser

Men who have sex with men using Pre-Exposure Prophylaxis show a high prevalence of anal precancerous lesions, similar to people with HIV, highlighting the need for targeted screening and further research on anal cancer prevention

## Graphical Abstract

**Graphical Abstract:**
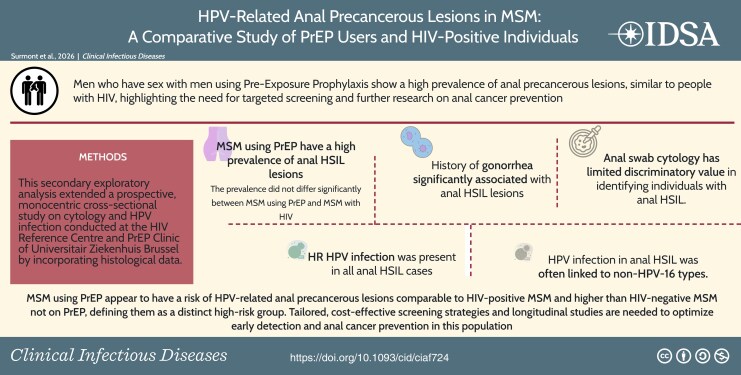
This graphical abstract is also available at Tidbit: https://tidbitapp.io/institutional-portal/clinical-infectious-diseases/tidbits/hpv-related-anal-precancerous-lesions-in-msm-a-comparative-study-of-prep-users-and-hiv-positive-individuals/update

Although anal Squamous Cell Carcinoma (SCC) is rare (1.7 per 100 000 person-years), it disproportionately affects high-risk populations, including people with HIV (PLH), men who have sex with men (MSM), solid organ transplant recipients and individuals with prior vulvar (pre-)cancer, showing at least a tenfold higher incidence [1].

Over 90% of cases are caused by persistent High-Risk Human Papillomavirus (HRHPV) infection. Cancer development follows a precancerous stage, known as anal High-grade Squamous Intraepithelial Lesions (HSIL) on histology. Risk factors for HPV infection, HSIL and thereby anal SCC include immunosuppression, receptive anal intercourse, smoking, early sexual debut and multiple sexual partners [2]. Coinfections such as gonorrhea, herpes simplex virus type 2, hepatitis B and syphilis may amplify HSIL risk [3].

MSM, both HIV-negative and HIV-positive, are a well-established high-risk group for anal cancer, warranting screening. Respectively 37–56% of HIV-negative and 75–86% of HIV-positive MSM have a HRHPV infection and 11% and 22%, respectively, have anal HSIL lesions [4–7].

The ANCHOR trial demonstrated that identifying and treating HSIL lesions reduced anal cancer incidence by 57% in PLH [8], underscoring the importance of HSIL detection and treatment in high-risk populations. Consequently, the International Anal Neoplasia Society (IANS) recommends routine screening for at-risk groups [1].

Current algorithms incorporate anal cytology and HRHPV testing on anal swabs (cytology alone; cytology + HRHPV triage; HRHPV alone; HRHPV + cytology triage; or co-testing), but no consensus exists on the optimal approach [9, 10]. Individuals with positive screening results are referred for High-Resolution Anoscopy (HRA). Efficient strategies must be identified to maximize HSIL detection, minimize unnecessary HRA referrals and avoid overburdening the healthcare infrastructure.

The introduction of HIV Pre-Exposure Prophylaxis (PrEP) created a distinct subgroup: HIV-negative MSM using PrEP (MSM-PrEP). These individuals, characterized by high-risk sexual behaviors such as condomless anal intercourse, multiple sexual partners and frequent sexually transmitted infections (STIs), are also vulnerable to anal HRHPV infection and anal precancerous lesions [11].

High HPV prevalence and abnormal cytology in MSM-PrEP, comparable to the prevalence in MSM with HIV (HIV + MSM), have been reported in studies conducted in Italy, France and China [11–14]. In France, anal HPV prevalence ranged from 92 to 93.4%, with HRHPV detected in 81.9–84% of participants. In China, overall anal HPV prevalence was 57.6% and HRHPV 33.6%. In Italy, any HPV in the anus was found in 87.2% and HRHPV in 79.2% of participants. Cytology was evaluated only in the French study, where 32% of anal swabs were normal, while 23% showed ASC-US, 40% LSIL, 5% HSIL, and 1% ASC-H, indicating that 68% had an abnormal cytology result. This aligns with the Belgian monocentric study by Surmont et al [6], which found similar rates of HRHPV infection (74.3% vs 75.3%) and abnormal anal cytology (53.5% vs 56.8%) between MSM-PrEP and HIV + MSM. Both groups showed higher rates compared to HIV-negative MSM not using PrEP. However, histological data, and thus prevalence of precancerous lesions, among MSM-PrEP remain lacking.

Study aims to

- Compare the prevalence and distribution of histologically confirmed anal HSIL (hHSIL) detected through HRA in MSM-PrEP compared to HIV + MSM
- Explore potential predictors for hHSIL, including age, smoking, chemsex, history of STIs and HPV vaccination status
- Assess the association between hHSIL and the anal swabs results (HRHPV infection and abnormal cytology) to better understand which approaches were effective in detecting lesions

## METHODS

### Study Design and Population

This secondary exploratory analysis extended a previous prospective, monocentric cross-sectional study conducted at the HIV Reference Centre and PrEP Clinic of Universitair Ziekenhuis Brussel. This prospective study included MSM presenting for routine follow-up consultations: HIV-uninfected MSM-PrEP and HIV + MSM, all aged ≥18 years. Exclusion criteria were recent perianal interventions (≤3 months), ongoing treatment for perianal HPV lesions or use of rectal enema, or receptive anal intercourse within 24 hours prior to sampling. All eligible participants provided a clinician-collected anal swab for HPV genotyping and cytology and completed a questionnaire covering behavioral and clinical risk factors.

Based on swab results, a subset of participants was referred for HRA with targeted biopsies when indicated. This analysis focuses on the histological outcomes among participants who underwent HRA. As this was a secondary analysis, no power calculation was performed.

### Data Collection

Between 2020 and 2023, anal samples were collected using a Rovers® Anex® Brush placed in ThinPrep vials containing PreservCyt® solution (Hologic, Inc.). Samples were tested for HRHPV (HPV16, HPV18 and pooled HR types 31, 33, 35, 39, 45, 51, 52, 56, 58, 59, 66, 68) using the COBAS® 4800 HPV test. Cytology was graded according to the Bethesda classification system as Negative for Intraepithelial Lesion or Malignancy (NILM), Atypical Squamous Cells of Undetermined Significance (ASC-US), Atypical Squamous Cells-cannot exclude HSIL (ASC-H), Low-grade Squamous Intraepithelial Lesion (LSIL) and High-grade Squamous Intraepithelial Lesion (HSIL).

Participants were informed of the results by phone. Those who met predefined referral criteria were scheduled for HRA. Referral criteria differed between the 2 groups and consisted of a co-testing approach: for HIV + MSM, any cytological abnormality (ASC-US or worse) and/or any HRHPV infection warranted referral; for MSM-PrEP, referral was based on ASC-US or worse and/or the presence of HPV16. Some participants requested HRA independently. Participants who declined or could not be reached were classified as lost to follow-up.

High-resolution anoscopy was performed at UZ Brussel between 15 December 2020 and 6 June 2024, by a single IANS-trained operator, using a Zeiss EXTARO 300 colposcope. Following application of 5% acetic acid, suspicious lesions were identified and biopsied using forceps. Biopsies at the squamocolumnar junction were performed without anesthesia and those below the dentate line were performed under local anesthesia. Biopsies were preserved in formalin and classified as normal (ie no LSIL or HSIL), LSIL or HSIL; p16 immunostaining confirmed HSIL. In cases of multiple lesions, the highest-grade histological diagnosis was recorded. If no lesions were visible and no biopsies were taken, participants were categorized as “no lesion”.

### Statistical Analysis

#### Prevalence of Histological Anal High-Grade Squamous Intraepithelial Lesion and Distribution of Histological Abnormalities

To estimate the prevalence of hHSIL, all participants who underwent HRA were included regardless of referral reason. hHSIL was defined as histopathologically confirmed HSIL on biopsy. Histological outcomes were categorized as no lesion, normal histology, LSIL or HSIL. hHSIL prevalence was calculated separately for MSM-PrEP and HIV + MSM, expressed as percentages with 95% binomial confidence intervals (CIs). Odds ratios (ORs) and 95% CIs compared hHSIL prevalence between groups; differences in the distribution of histological outcomes were tested using the Mann–Whitney *U* test.

#### Exploratory Analysis of Predictors for Histological High-Grade Squamous Intraepithelial Lesion

Univariate logistic regression was performed in the subgroup of all patients who had undergone HRA to explore associations between outcome hHSIL and potential predictors: age; smoking (yes/no); chemsex (yes/no); the presence of history of specific STIs, namely, gonorrhea, chlamydia and syphilis (each recorded as a binary yes/no variable, based on self-report and/or medical records); and HPV vaccination (yes/no). Each variable was analyzed separately, with results expressed as OR with corresponding 95% CI.

#### Statistical Analysis of Anal Swab Results in Relation to Histological High-Grade Squamous Intraepithelial Lesion Detection

To explore the relationship between hHSIL and anal swab results, univariate logistic regression analyses were performed using HRHPV results and cytology as independent variables. The total number of hHSIL cases was used to retrospectively evaluate how many would have been detected if referral had relied solely on HRHPV testing or cytology.

All statistical analyses were performed in Stata (version 13, StataCorp, USA), with statistical significance set at *P* < .05.

This study was approved by the Ethical Committee of UZ Brussel (EC 2024/207).

## RESULTS

### Sample Description

Patients were referred for HRA after anal swab screening. In this initial prospective study, a total of 298 MSM participated: 148 MSM-PrEP and 150 HIV + MSM.

Among MSM-PrEP, 88/148 (59.5%) met referral criteria: ASC-US or higher (77/148, 52%) and/or HPV16 positivity (44/148, 29.7%). Of these, 77 (87.5%) underwent HRA, with 12.5% lost to follow-up and 8 MSM-PrEP subjects requested HRA outside the protocol. In total, 85/148 (57.0%) MSM-PrEP underwent HRA.

Among HIV + MSM, 118 out of 150 (78.7%) had an HRA indication: ASC-US or higher (83/150, 55.3%) and HRHPV (113/150, 75.3%). Of these, 92 (78.0%) underwent HRA, while 26 (22.0%) were lost to follow-up. One participant requested HRA independently. In total, 93/150 (62.0%) HIV + MSM underwent HRA.

### Prevalence of Histological Anal High-Grade Squamous Intraepithelial Lesion and Distribution of Histological Abnormalities Detected via High-Resolution Anoscopy in Men Who Have Sex With Men Using Pre-exposure Prophylaxis Compared With Men Who Have Sex With Men with HIV

**Figure 1.**
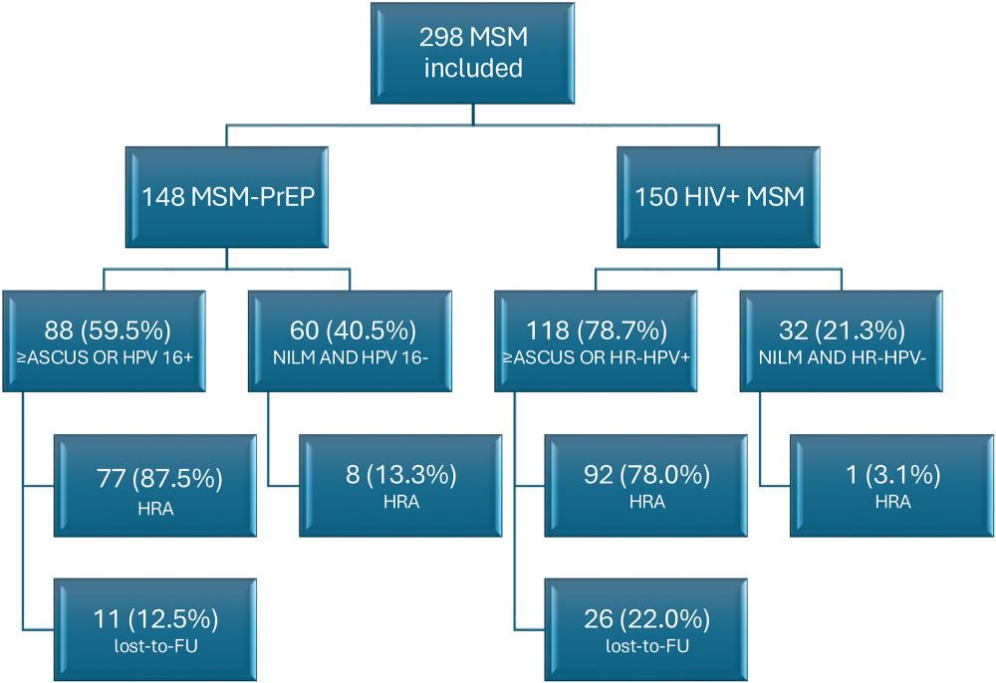
Flowchart of the patients included in the analysis.

In total, 85 MSM-PrEP and 93 HIV + MSM underwent HRA (Figure 1). Biopsies were taken in 67 (78.9%) MSM-PrEP and 72 (77.4%) HIV + MSM.

hHSIL was the worst histological finding in 19 (22.4%) MSM-PrEP and 29 (31.2%) HIV + MSM (Tables 1 and 2). The OR for hHSIL in HIV + MSM compared to MSM-PrEP was 0.6 (95% CI .32–1.25) (Table 1). The distribution of histological results (HSIL, LSIL, normal, no lesion) did not differ significantly between the 2 groups (*P* = .401) (Table 2).

**Table 1.** Factors Associated With hHSIL Among MSM-PrEP and HIV + MSM Who Underwent HRA, Belgium, 2020–2024

	All Study Participants		Participants Without hHSIL		Participants With hHSIL			
All MSM with HRA	178	(100.0)	130	(73.0)	48	(27.0)	Crude OR for hHSIL (95% CI)	
Age (mean in y, SD)	45.4	(11.2)	45.5	(11.2)	45.4	(11.2)	1.00	(0.97–1.03)
Study group								
MSM-PrEP	85	(47.8)	66	(50.8)	19	(60.4)	Ref.	…
HIV + MSM	93	(52.2)	64	(49.2)	29	(39.6)	0.64	(0.32–1.25)
Tobacco consumption								
Never smoker	105	(59.0)	75	(57.7)	30	(62.5)	Ref.	…
Former smoker	22	(12.4)	14	(10.8)	8	(16.7)	1.43	(0.54–3.75)
Current smoker	51	(28.6)	41	(31.5)	10	(20.8)	0.61	(0.27–1.37)
Chemsex ≥ 1 time in last 4 wks	31	(17.4)	21	(16.2)	10	(20.8)	1.37	(0.59–3.16)
STIs								
Had syphilis ≥ 1 time	67	(37.6)	50	(38.5)	17	(35.4)	1.14	(0.57–2.27)
Had chlamydia ≥ 1 time	43	(24.2)	35	(26.9)	8	(16.7)	1.84	(0.78–4.31)
Had gonorrhea ≥ 1 time	50	(28.1)	42	(32.3)	8	(16.7)	2.39	(1.03–5.55)
HPV vaccination	16	(9.0)	13	(10.0)	3	(6.2)	0.6	(0.16–2.21)

Abbreviations: HIV, human immunodeficiency virus; HPV, human papilloma virus; HRA, high resolution anoscopy; HSIL, high-grade squamous intraepithelial lesions; MSM, men who have sex with men; PrEP, pre-exposure prophylaxis, SD, standard deviation; STI, sexually transmitted disease.

Data are N (%) unless stated otherwise.

**Table 2.** Distribution of Different Anal Histology Among MSM-PrEP and HIV + MSM Who Underwent HRA, Belgium, 2020–2024

	MSM-PrEP			HIV + MSM			
	N	(%)	[95% CI]	N	(%)	[95% CI]	*P* Value
Histology							.401
HSIL	19	(22.4%)	[21.4–23.4]	29	(31.2%)	[29.7–32.3]	
LSIL	31	(36.5%)	[35.3–37.7]	25	(26.9%)	[25.7–28.3]	
Normal	17	(20.0%)	[19.0–21.0]	18	(19.5%)	[17.7–20.3]	
No lesion	18	(21.2%)	[20.2–22.2]	21	(22.6%)	[21.6–23.6]	

Abbreviations: HIV, human immunodeficiency virus; HRA, high resolution anoscopy; HSIL, high-grade squamous intraepithelial lesions; LSIL, low-grade squamous intraepithelial lesion; MSM, men who have sex with men; PrEP, pre-exposure prophylaxis.

#### Exploratory Analysis of Other Predictors for Histological Anal High-Grade Squamous Intraepithelial Lesion

A history of gonorrhea was significantly associated with hHSIL (OR 2.39; 95% CI 1.03–5.55). Other STIs, smoking and chemsex were not significantly associated but showed similar trends. Human papillomavirus vaccination was associated with a lower hHSIL prevalence, though not statistically significant (Table 1).

#### Analysis of Anal Swab Results in Relation to Histological Anal High-Grade Squamous Intraepithelial Lesion Detection

**Figure 2.**
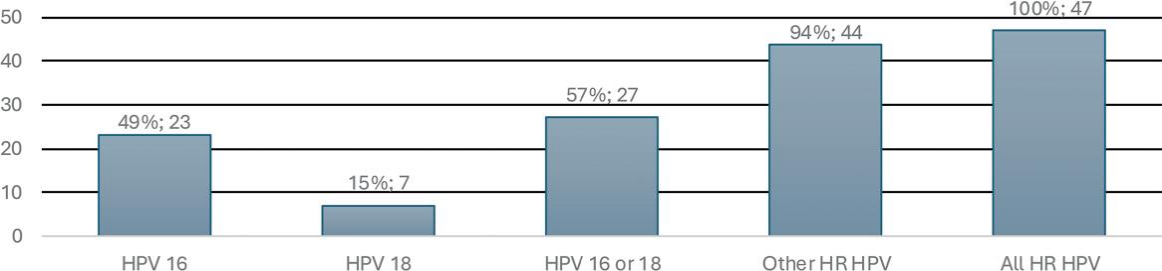
Distribution of HRHPV types in histological anal HSIL (N = 47) among MSM-PrEP and HIV + MSM who underwent HRA, Belgium, 2020–2024. Abbreviations: HIV, human immunodeficiency virus; HRA, high resolution anoscopy; HRHPV, high-risk human papillomavirus; HSIL, high-grade squamous intraepithelial lesion; MSM, men who have sex with men; PrEP, pre-exposure prophylaxis.

Using a co-testing referral strategy, 47 hHSIL cases were identified among 169 referred participants. One hHSIL was detected after participant-requested HRA. Restricting referral to HPV16 positivity would have missed 24/47 hHSIL cases (51%); HPV18 alone would have missed 40/47 hHSIL cases (85%). Combined HPV16/18 would still have missed 20 hHSIL lesions (43%). Targeting other HRHPV types, excluding HPV16 and 18, would have detected 94% (44/47) of hHSIL, missing only 3 cases (6%). HRHPV infection was present in all hHSIL cases (47/47) (Figure 2). ORs for hHSIL by HPV type showed a trend toward stronger association for HRHPV types other than 16 or 18 (OR 3.08) compared to HPV16 (OR 1.63) and HPV18 (OR 1.23) (Table 3).

**Table 3.** Association Between Anal Swab Results in Relation to hHSIL Among MSM-PrEP and HIV + MSM Who Underwent HRA, Belgium, 2020–2024

	All Study Participants		Participants Without hHSIL		Participants With hHSIL		Crude OR For hHSIL (95% CI)	
All MSM who underwent HRA	178	(100.0)	130	(73.0)	48	(27.0)	…	…
HRHPV on anal swab								
HPV16^a^ on swab (2 missing)	71	(40.3)	48	(37.2)	23	(48.9)	1.62	(0.82–3.17)
HPV18^a^ on swab (1 missing)	26	(14.7)	18	(14.0)	8	(16.7)	1.23	(0.49–3.06)
Other HRHPV^a^ on swab (1 missing)	152	(85.9)	107	(83.0)	45	(93.7)	3.08	(0.87–10.82)
≥1 HRHPV on swab	168	(94.9)	120	(93.0)	48	(100.0)	…	…
Cytology on anal swab								
NILM	39	(21.9)	32	(24.6)	7	(14.6)	Ref.	…
LSIL	37	(20.8)	25	(19.2)	12	(25.0)	2.19	(0.75–6.39)
ASC-US	80	(41.7)	60	(46.2)	20	(41.7)	1.52	(0.58–3.99)
ASC-H	17	(12.5)	11	(8.5)	6	(12.5)	2.49	(0.69–9.03)
HSIL	5	(6.2)	2	(1.5)	3	(6.2)	6.85	(0.96–49.03)

Abbreviations: ASC-H, Atypical Squamous Cells—cannot exclude HSIL; ASC-US, Atypical Squamous Cells of Undetermined Significance; hHSIL, histologically confirmed anal HSIL; HIV, human immunodeficiency virus; HRA, high resolution anoscopy; HRHPV, high-risk human papillomavirus; HSIL, high-grade squamous intraepithelial lesions; LSIL, low-grade squamous intraepithelial lesion; MSM, men who have sex with men; NILM, Negative for Intraepithelial Lesion or Malignancy; PrEP, pre-exposure prophylaxis.

Data are N (%) unless stated otherwise.

^a^Possibly also positive for other HRHPV.

**Figure 3.**
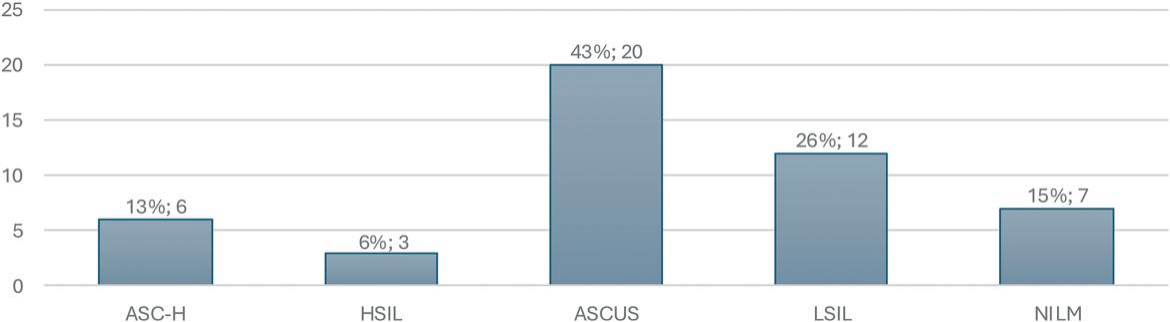
Distribution of cytological results in histological anal HSIL (N = 47) among MSM-PrEP and HIV + MSM who underwent HRA, Belgium, 2020–2024. Abbreviations: HIV, human immunodeficiency virus; HRA, high resolution anoscopy; HSIL, high-grade squamous intraepithelial lesion; MSM, men who have sex with men; PrEP, pre-exposure prophylaxis.

Among hHSIL lesions, cytology showed ASC-US in 43%, LSIL in 26% and NILM, ASC-H and HSIL in 15%, 13% and 6%, respectively (Figure 3). hHSIL risk increased with cytological severity: compared with normal cytology, ORs were 2.19 for LSIL and 6.85 for HSIL. The largest proportion of hHSIL had ASC-US cytology, while LSIL and NILM cytology together accounted for approximately 40% of cases.

## DISCUSSION

### Histological Anal High-Grade Squamous Intraepithelial Lesion Prevalence and Histological Distribution

Our findings show that hHSIL was common in both MSM-PrEP and HIV + MSM with no statistical difference in histological distribution between the 2 groups (Table 2). Although evidence on MSM-PrEP remains scarce, the hHSIL prevalence in this group clearly exceeds the 11% reported in HIV-negative MSM [4], suggesting that MSM-PrEP may represent a subgroup with an increased susceptibility to anal precancerous lesions. Similarly, the hHSIL prevalence observed among HIV + MSM was higher than the 22% reported in the literature [4]. These high prevalences in both groups may reflect selection bias, as participants referred for HRA constitute a subgroup enriched with individuals at higher risk.

### Predictors for Histological Anal High-Grade Squamous Intraepithelial Lesion

A history of gonorrhea was the only variable significantly associated with hHSIL. This finding is consistent with previous evidence suggesting a potential biological synergy between HPV and other STIs, including the identification of gonorrhea as a risk factor for HPV acquisition among HIV + MSM [2]. However, HPV infection alone does not necessarily lead to progression toward precancerous lesions. McCloskey et al [3] found a similar association between gonorrhea and hHSIL, supporting the hypothesis of synergistic interactions between HPV and other STIs. Given that PrEP use is associated with increased STI incidence, these findings raise concern about a possible indirect effect of PrEP-related sexual behavior on anal oncogenic risk [2]. Further research is needed to clarify long-term oncogenic risks in MSM-PrEP, particularly in the context of STI history.

### Anal Swab Results and Screening Implications

Similar to standard care, referral decisions in this study were based on anal swab results. The predefined referral criteria were intentionally stricter for HIV + MSM, reflecting their higher baseline risk for anal cancer. HIV-positive participants were referred when any HRHPV type was detected, whereas MSM-PrEP were referred only when HPV16 was present, consistent with its predominant role in anal cancer cases among HIV-negative populations.

Screening performance for anal precancer varies considerably across studies, particularly for cytology. Cytology alone is generally not recommended because of its limited sensitivity in high-risk MSM. Our data support this limitation: hHSIL was observed across the full range of cytological categories, including cases with normal cytology (Figure 3). Although cytology showed a gradual increase in hHSIL risk with increasing abnormality, the overlap between categories suggests that it lacks sufficient discriminatory value for reliable triage (Table 3).

Human papillomavirus testing alone demonstrated higher sensitivity (96% vs 86%) and a higher negative predictive value (91% vs 76%) than cytology, but specificity remained low (29%) in populations with a high HRHPV prevalence [10]. Our findings underline the contribution of non-HPV16 HR types to hHSIL, indicating that reliance on HPV16-based referral strategies alone, such as the French clinical practice recommendations [15], would likely miss a relevant proportion of lesions (Figure 2). Conversely, referring all HRHPV-positive individuals may not be feasible because of the potential strain on clinical capacity.

At present, no single screening strategy emerges as clearly optimal for hHSIL detection among MSM. Recent work suggests that molecular biomarkers, particularly methylation-based assays, may offer improved risk stratification by reducing unnecessary referrals while maintaining adequate sensitivity [16]. Incorporating such molecular biomarkers may improve detection performance with clinical feasibility, though longitudinal studies are needed to determine their effectiveness in routine practice.

### Strengths

This is the first study to document hHSIL in MSM-PrEP, as previous research has focused primarily on HRHPV infections and cytological abnormalities. The availability of clinical and behavioral data also enabled exploratory analysis of potential predictors of histological anal HSIL. Together, these features strengthen the study's contribution to refining risk-based screening and prevention strategies for MSM.

### Limitations

As this is a secondary analysis, no power calculation was performed for the specific research questions. The sample size may have been insufficient to detect significant differences or to confirm equivalence between groups. The predefined referral criteria limit the ability to estimate diagnostic performance metrics, since not all participants underwent HRA. As such, true disease status is unknown for non-referred individuals, introducing potential verification bias. Furthermore, associations between hHSIL and swab-based predictors were evaluated only within the subset referred for HRA, representing a preselected high-risk group. Reported ORs therefore reflect within-group associations within this enriched group rather than population-level risk. Despite these limitations, these findings underscore the need for standardized prospective studies to clarify anal hHSIL risk in MSM-PrEP.

## CONCLUSION

MSM-PrEP appear to have a risk of HPV-related anal precancerous lesions similar to HIV + MSM and higher than HIV-negative MSM not using PrEP. This positions MSM-PrEP as a potential distinct high-risk group for HPV-related anal neoplasia for whom current screening guidelines may be insufficient. Defining their risk profile is essential to guide targeted screening, improve early detection and reduce the likelihood of underdiagnosing high-grade anal lesions in the future. Longitudinal studies incorporating novel biomarkers and cost-effective, risk-adapted screening strategies are warranted to strengthen anal cancer prevention in MSM populations.
